# Evaluation of Prescription Patterns of Antipsychotics in Schizophrenia Patients—A Single-Center Prospective Study

**DOI:** 10.3390/jcm14092941

**Published:** 2025-04-24

**Authors:** Ahmed Adel Mohamed, Abdulaziz Saleh Almulhim, Abdulrahman Abdullah Alnijadi, Fatimatuzzahra’ binti Abd Aziz, Khuloud Khaled Alajmi, Ahmed Abdullah Al-Mudhaffar, Mohammad Salem Almutairi

**Affiliations:** 1 Department of Clinical Pharmacy, School of Pharmaceutical Sciences, Universiti Sains Malaysia, Penang 11800, Malaysia; ahmedadelm@student.usm.my; 2 Department of Pharmacy Practice, College of Clinical Pharmacy, King Faisal University, Al Ahsaa 31982, Saudi Arabia; aalnijadi@kfu.edu.sa; 3 Al Ahsaa Psychiatric Hospital, Al Ahsaa 36445, Saudi Arabia; kalajmi@moh.gov.sa (K.K.A.); malmutairi13@moh.gov.sa (M.S.A.)

**Keywords:** schizophrenia, polypharmacy, maximum dose, antipsychotics, inappropriate use

## Abstract

Inappropriate prescription patterns and polypharmacy are critical challenges facing the optimal management of schizophrenia patients, especially in regard to patient safety. **Background/Objectives**: The purpose of this study was to examine the relationship between patient safety and the existence of incorrect prescription patterns and/or polypharmacy in the medications prescribed to individuals with schizophrenia. This issue is addressed in a broad context, highlighting the purpose of this study. **Methods**: A cross-sectional study was adopted, involving a prospective analysis of the prescriptions of schizophrenia patients receiving treatment. Prescription patterns deemed inappropriate were evaluated based on evidence-based guidelines. Antipsychotic maximum allowable daily doses were calculated using the British National Formulary Maximum Daily Dose (BNFmax), an online tool. Patient safety outcomes were assessed using the Glasgow Antipsychotic Side-effect Scale (GASS). **Results**: A total of 198 patients diagnosed with schizophrenia and receiving treatment consented to participate in the GASS survey. A total of 116 (58.6%) males participated. The mean age of patients was 40.1 (±12.7). Thirty-one (66.2%) reported mild side effects, while 67 (33.8%) reported moderate side effects. Polypharmacy was detected in 103 (52%) patients’ prescriptions. The correlation between GASS and BNFmax was positive and statistically significant (*p* < 0.001). The elevation in GASS score was associated with polypharmacy prescriptions (OR 3.21; 95% CI 1.64–6.29), the presence of first-generation antipsychotics (FGAP) (OR 2.79; 95% CI 0.236–5.951), any combination of antipsychotics containing haloperidol (OR 3.22; 95% CI 1.11–9.32), and olanzapine (OR 3.46; 95% CI 1.36–8.79). **Conclusions**: The safety of patients with schizophrenia has been proven to be impacted by the improper use of psychotropic drugs. Following evidence-based guidelines is a cornerstone to ensuring optimal, effective, and safe patient treatment plans.

## 1. Introduction

One of the most incapacitating mental illnesses is schizophrenia. It typically starts early in life and significantly impairs the patient’s functionality, productivity, and social activities [1]. Achieving schizophrenia therapeutic goals is a challenging step due to many factors related to the disease itself and the available treatment options. Schizophrenia has variable forms of symptoms. The psychotic or positive domain includes delusions, hallucinations, disorganized behavior, and incomprehensible speech. The negative symptoms include a lack of spontaneity, reduced energy and speech, apathy, and anhedonia. The third domain includes cognitive symptoms, which consist of a wide range of impairments of different cognitive skills [2]. Positive signs of schizophrenia usually show up as a pattern of relapses and remissions. Few patients endure more extended psychotic features. Negative and cognitive symptoms are more persistent and have a long-term impact on patient social functioning [3].

Since schizophrenia is a chronic condition, treatment objectives should focus on long-term results, such as preventing relapses and avoiding adverse physical morbidity and mortality effects from a prolonged antipsychotic load. These are crucial issues for the patient’s well-being. Antipsychotic (AP) medications are the cornerstone pharmacotherapy approved for the management of schizophrenia. Two groups of AP are available [4]. The first-generation antipsychotics (FGAP), known as the conventional or typical group, mainly target positive symptoms but cause movement disorders and tardive dyskinesia. Atypical antipsychotics, often known as second-generation antipsychotics (SGAP), can influence both positive and negative symptoms. However, they cause fewer movement disorders but lead to more metabolic adverse effects, especially in high doses and in combination use [5,6].

Despite the reasonable control that AP generally offers to the patient’s symptoms, some patients develop permanent negative features. This stage of the disease is known as the deficit syndrome, and its prevalence is reported as 15–20% [7]. The clinical features of the negative symptoms have many similarities with depression. Since the full course of traditional antipsychotic treatment does not alleviate these persistent negative symptoms, many doctors prescribe antidepressants [8]. According to the American Psychiatric Association practice guidelines (APA 2021), using adjunctive antidepressant medications may be considered in this case because of their relative safety. However, mood stabilizers (sodium valproate or lamotrigine) are added to some selected cases in clinical practice based on the resistance to the positive symptoms to control the patient’s neuronal hyper-excitability combined with aggression [9]. The available evidence does not sufficiently support this practice’s efficacy and safety [1].

Antipsychotics are frequently linked to a variety of undesirable side effects. Fifty to seventy percent of patients experience at least one side effect [10]. This high adverse effect rate affects the patient’s attitude toward the treatment plan, causing practitioners to design a treatment plan based on tolerability rather than desired efficacy. Guidelines frequently recommend selecting antipsychotic drugs based on side effect profiles rather than effectiveness [11,12]. The adverse effects vary in severity between patients. Some adverse effects are tolerable (e.g., light drowsiness or dry mouth), some are markedly unpleasant (e.g., resistant constipation, sexual dysfunction, and movement disorders), some are painful (e.g., acute dystonia), and some even pose a threat to life (e.g., neuroleptic malignant syndrome, agranulocytosis, and myocarditis) [12,13,14,15,16,17]. Metabolic side effects (weight gain, hypercholesterolemia, hyperglycemia, and increased prolactin levels) elevate the incidence of other complications, such as diabetes mellitus and cardiovascular diseases, by 50% [13].

Polypharmacy is defined as using multiple medications to treat a disease condition without clinical indication and supporting evidence [18,19]. Medications that treat adverse effects secondary to polypharmacy are also considered polypharmacy [20]. According to the APA and Maudsley prescribing guidelines [11,21], patients should only be prescribed a single antipsychotic. If the treatment fails, switching to another one from the same or a different group is recommended. After switching two to three times without symptom improvement, clozapine-based therapy can be considered. Both guidelines do not recommend any combination of antipsychotic agents. In some special situations, a time-limited combination with clozapine may be considered. Many recent reviews reported that the evidence supporting polypharmacy therapy with schizophrenia is weak, with a significant elevation of the likelihood of side effects. This should be carefully monitored and considered when this practice is performed [1,22].

A nationwide cohort study in Finland found that only a few antipsychotic combinations should be considered because of their effect on decreasing the rate of rehospitalization [23]. This cohort did not perform a precise evaluation of the safety of those combinations, so a more profound safety measuring assessment would be helpful in guiding the selection of the safest antipsychotic combinations when prescribed.

In clinical practice, the rate of polypharmacy reported in many studies ranges from 20–66% [24,25,26]. However, there is not enough substantial evidence to support this practice. Furthermore, polypharmacy raises the patient’s risk of mortality, relapse, falls, bad drug reactions, and extended hospital stays [27,28,29]. Based on the currently available evidence, the hazards and advantages of polypharmacy with schizophrenia patients are conflicting. Furthermore, it is unknown how polypharmacy influences the occurrence of adverse medication events in schizophrenia patients.

The Royal College of Psychiatrists developed a tool for measuring the dosing appropriateness of AP called BNFmax, which stands for the British National Formulary maximum allowable daily dose of the prescribed AP. This tool was developed based on a recently released updated report (2014; January 2023 revision). Using this tool, the appropriateness of the total daily dose of single or combined AP a patient receives can be determined. According to the Royal College of Psychiatrists, a dose of AP prescription is considered high if the total daily dose of a single agent or a combination of percentages exceeds the BNFmax by 100%. This tool is used widely in the literature to demonstrate the dosing appropriateness of AP [30,31,32]. According to reports, keeping patients on high dosages increases the risk of adverse medication events, decreases patient compliance, increases resistance to treatment, and results in poor pharmacological response [12,33].

In Saudi Arabia, recent studies described some indicators of inappropriate use of AP with schizophrenia patients. According to these studies, polypharmacy was found in 42.3% of prescriptions [34]. In addition, other studies reported a tendency to prescribe high doses of AP [35,36]. No study correlates these inappropriate practices to the direct impact on patients. In addition, none of them explored the possible factors that may be leading to this improper use. These findings could help in reducing the impact on schizophrenia patients.

The purpose of this study was to assess the AP prescribing patterns among individuals with schizophrenia and the association between these practices and the increased probability of adverse effects on patients.

## 2. Materials and Methods

### 2.1. Ethical Considerations:

Every procedure used in this study complied with the 2008 revision of the Helsinki Declaration of 1975 and the ethical guidelines set out by the relevant national and institutional human experimentation committees. The local committee of the King Fahd Hospital-Hofuf Institutional Review Board examined and approved this study’s protocol (IRB KHFF No. H-05-HS-065).

### 2.2. Study Design

This was a cross-sectional, patient-centered, self-reported study targeting the inpatient and outpatient departments of Al-Ahsaa Psychiatric Hospital from (February to August 2023). Al-Ahsaa Psychiatric Hospital is a 200-bed governmental institution serving 1.1 million people [37]. It is one of two psychiatric hospitals in Saudi Arabia’s Eastern Province.

All inpatients and outpatients in the hospital were evaluated for eligibility. Patients older than 18 diagnosed with schizophrenia, who had been prescribed at least one of the psychotropic medications (antipsychotics, antidepressants, or mood stabilizers), started a treatment plan for one month or more, and were capable of understanding and signing the informed consent were included. The patient’s demographics, diagnosis, list of prescribed medications, and laboratory investigation data were collected. Patients with diagnoses of any other mental illness (bipolar disorder, depression, and anxiety) and/or if there was any evidence that the patient’s condition was secondary to drug abuse or any illegal drug use were excluded.

Eligible patients were invited to participate in this study by completing a self-reported validated Arabic version of the Glasgow Antipsychotic Side Effects Scale (GASS) [38].

### 2.3. Assessment Tools

#### 2.3.1. Glasgow Antipsychotic Side-Effect Scale (GASS)

GASS is a validated instrument designed for screening and monitoring adverse effects in patients using AP. The uniqueness of this instrument is that it could be used to assess both neurological and non-neurological side effects. It is designed mainly for clinical practice settings [39,40].

In short, GASS is a self-reported questionnaire that the patient can fill out alone or with assistance from the healthcare team. It comprises 22 items, each assessed by a four-point Likert scale (Never, Once, A few times, or Every day).

#### 2.3.2. Prescription Assessment

After the patient fulfilled the requirements for inclusion and signed the informed consent form, all prescription data were gathered. No private patient data were collected; only the patient file number was registered in a private sheet with the primary study investigator to avoid any data duplication, and these data were deleted entirely after finishing the collection. The following data were collected from the patient files: diagnosis, sex, age, any other comorbidities, years since the initial diagnosis, and all prescribed medications with doses, dosage forms, and number of daily doses. Medication compliance was assessed by asking patients directly as well as counting missing doses from previous refills.

Polypharmacy was defined as two or more psychotropics prescribed for one month or more [18]. However, in the current study, we did not rely solely on the numerical definition of polypharmacy, as this study’s primary goal was to determine the appropriateness of the prescribed medications. Therefore, we focused on the clinical judgment of the combination treatment based on patient safety and concordance with the evidence-based guidelines [41,42]. Bearing in mind the relapsing/remitting pattern of schizophrenia, patients who received AP as needed or during the switching stage between two different APs were excluded. Additionally, it was not considered polypharmacy if the patient was given an add-on antidepressant or mood stabilizer to a single antipsychotic, as the effect of this combination was supported by some studies [19]. The appropriateness of dose titration was assessed by referring to the patient files during the initiation of the AP treatment if they were starting directly on a high AP dose or following the recommended scheduled dose increase defined by guidelines and the UpToDate drug information monograph [11,43].

We used the British National Formulary maximum allowable daily dose of the prescribed AP (BNFmax) indicator. This assessment tool is based on counting all the prescribed oral and long-acting AP doses and frequency and giving a percent for each AP (in case of polypharmacy); the summation indicates the total daily AP for the patient. In this study, we used an online calculator (https://kornor.github.io/, accessed on 1 February 2023) that facilitated the process of BNFmax dose calculations.

### 2.4. Sampling Method

The number of prescriptions that the pharmacy department dispensed was used to determine the sample size, with a total of 4000 per month. Patients were randomly selected over the data collection period until the targeted calculated sample size was reached. To calculate the required sample size, we used the single proportion formula because the main measurable factor in this study is the percentage of adverse drug reactions reported by the patients [44]. We assumed the confidence level value (1.96) to find a 95% confidence interval and 5% margin of error with a 24% population proportion adopted from two previous studies [45,46]. According to the calculations mentioned above, the required sample was 196.

### 2.5. Statistical Methods

The study variables were analyzed using a descriptive analysis. Each categorical variable’s median and interquartile range (IQR) were shown as percentages and figures. Regarding the continuous variables, after confirming the normality with the Shapiro–Wilk Test and rolling out the skewness of these data, these data were represented by mean and standard deviation. Binary logistic regression analysis was used to determine any associations between the main study dependent variables (having a high/moderate GASS score and having a BNFmax score of 100% or more), patients’ demographics, and prescription inappropriateness indicators. Sperman’s correlation was used to find the correlation coefficient between GASS and BNFmax. IBM SPSS Statistics for Windows, version 26.0 (IBM Corp., Armonk, NY, USA), was used to conduct the statistical study.

## 3. Results

A total of 253 patients were evaluated for eligibility, of which 198 met the inclusion criteria. One hundred sixteen participants were males (58.5%), and the average age was 40.1 (±121.7) years old. The history of schizophrenia illness was more than ten years for 51.01% of the participants (*n* = 111). Twenty-seven (13.6%) participants were diabetic patients, and 31 (15.7%) were hypertensive patients. Regarding the prescribed APs per patient, half received only one AP (*n* = 100, 50.51%), and seventy-nine (39.9%) received two APs. A number of prescriptions contained three and four APs (*n* = 16, 8.08%) (*n* = 3, 1.52%), respectively. FGAPs were found in 55 (27.8%) prescriptions, and SGAPs were prescribed for 181 (91.4%) patients. Polypharmacy was detected in 103 (52%) prescriptions. The BNFmax dose calculator results showed a mean value of 0.75 (±0.49). BNFmax was represented as categories: prescriptions with BNF ≤ 0.5 (*n* = 85, 42.9%); from more than 0.5 to less than one (*n* = 28.8, 28.8%); and almost the same number of prescriptions with BNFmax were more than or equal to one. The mean of overall GASS was 16.9 (9.3%). Out of 198 patients, 131 (66.2%) were categorized as having mild/moderate side effects, and 67 (33.8) had moderate/high side effects (Table 1).

All the APs prescribed for this study’s patients included twelve drugs, seven from SGAP and five from FGAP. Risperidone was the most frequently prescribed drug (*n* = 72, 36.36%), which is the most ordered for monotherapy as well (*n* = 31, 15.7). Olanzapine was the second most commonly prescribed AP (*n* = 70, 35.35%), while it was the most often used in AP combination (*n* = 43, 21.7%). The third was haloperidol, which was the only FGAP present in the highly prescribed list (*n* = 40, 20.7%). Out of these 40 prescriptions, *n* = 28, 14.1% were in combination with other APs. This was followed by aripiprazole (*n* = 36, 18.18%), quetiapine (*n* = 33, 16.67%), and paliperidone (*n* = 32, 16.16%) (Table 2).

Sedation was the most reported adverse effect (*n* = 117, 59.1%), followed by polyuria/polydipsia (*n* = 113, 57%), asthenia, and tremor (*n* = 97, 49%), and (*n* = 96, 48%) stated having weight gain (Table 3). According to the correlation analysis, the GASS score and the BNFmax have a strong positive association with the Pearson correlation coefficient of (0.250) at a significance level of less than (0.001).

Table 4 represents factors associated with having a moderate/high GASS score. The odds ratio for polypharmacy was 3.21 (95% CI 1.64–6.29), the total number of medications per prescription was 1.67 (95% CI 1.18–2.36), and the number of FGAP per prescription was 2.79 (95% CI 1.11–7.01).

The results of Binary Logistic regression to find the factors associated with exceeding 100% of BNFmax. (Table 5). The results showed the odds ratio for polypharmacy prescriptions 10.54 (95% CI 4.80–23.17), FGAP 5.46 (95% CI 2.44–12.20), SGAP 7.38 (95% CI 4.80–23.169, *p* < 0.001), and for the prescription containing at least one psychotropic drug did not initiate with appropriate dosing titration was 3.687 (95% CI 1.648–8.249, *p* = 0.001).

A logistic regression analysis for having a moderate/high GASS score being prescribed in different APs was performed to determine any association between using different APs as monotherapy or in combination. This analysis focused on the five most highly prescribed APs (according to Table 2). Our analysis revealed that using APs as a monotherapy was not associated with a high GASS score. The analysis showed a significant influence of the presence of two APs in any treatment combination. The odds ratios were 3.46 (95% CI 1.36–8.79) for olanzapine and 3.22 (95% CI 1.11–9.32) for haloperidol.

The same analysis found an association between the presence of different AP combinations included in prescriptions with BNFmax more than or equal to 100%. The odds ratio of paliperidone was 15.39 (95% CI 3.58–66.13), olanzapine 4.13 (95% CI 1.39–12.34), aripiprazole 7.48 (95% CI 1.83–30.55), and haloperidol 3.69 (95% CI 1.12–12.18) (Table 6).

## 4. Discussion

In this observational study, we evaluated the relationship between the likelihood of experiencing adverse effects and inappropriate AP usage in a large cohort of patients with schizophrenia. A clear correlation was observed between improper AP utilization and the likelihood of adverse outcomes. Concerning GASS results, polypharmacy, the total number of medications per prescription, and the number of FGAP per prescription were associated with high scores. With regard to BNFmax score, polypharmacy, use of FGAP and SGAS, and inappropriate AP dose titration were associated with elevated scores.

The majority (52%) of our cohort had polypharmacy. This rate is higher than in studies conducted in other areas, such as North America (16.1%), Europe (23%) [24], China (44.1) [47], and Japan (43%) [48]. Data are conflicting regarding the relationship between polypharmacy and an increase in side effects. Some studies found no relation between polypharmacy and an increasing rate of side effects [49,50], whereas other studies reported undesirable side effects secondary to the initiation of polypharmacy [51,52]. A recent nationwide cohort recommended performing more trials to confirm the actual impact of polypharmacy compared to monotherapy [53]. In our study, patients with polypharmacy had three times the odds of having moderate to high GASS scores.

Concerning the BNFmax score, patients with polypharmacy had ten times the odds of having a high BNFmax score. Analogizing our research, Corrado et al. conducted a prospective study to assess the prescribing pattern of AP medications [54]. Polypharmacy was the strongest independent variable associated with high AP doses. Moreover, the inappropriate dosing titration of the AP dose was another factor that was associated with a high BNFmax score, which may explain the elevation of side effect severity for this group of patients. Vadiei and colleagues conducted a retrospective study to assess AP dose titration patterns in inpatient settings. Interestingly, they recommended at least three days of dose titration [43]. Takeuchi et al. conducted a meta-analysis comparing rapid vs. slow AP titration. Although no significant difference was found between the two modalities regarding efficacy or intolerability, results should be interpreted cautiously due to clinical and statistical heterogeneity [55]. It is worth noting that the minimum duration in the included studies was six days.

In contrast to previously published studies, the percentage of prescriptions containing three or more psychotropic medications in our research was much higher (9.6%) (Table 1); this can explain the severity of adverse events in our studied sample [56,57,58]. A recent meta-analysis emphasized the need to consider simpler prescriptions by decreasing the number of medications and adjusting the dosing frequencies and medication doses [59].

In the current study, the analysis of the most commonly prescribed antipsychotic medications alongside the maximum recommended dosages outlined in the British National Formulary (BNF) sheds light on several critical issues. Notably, with the exception of risperidone and quetiapine, all other antipsychotic medications prescribed in the context of polypharmacy were significantly correlated with instances of exceeding the total daily dose limits established by the BNF. This pattern was consistent with findings from prior research, underscoring a prevalent challenge in clinical practice, particularly when clinicians opt to augment treatment regimens with an additional antipsychotic [30,54]. Such practices raise concerns regarding patient safety and the potential for increased adverse effects, emphasizing the need for careful monitoring and adherence to dosage guidelines in the management of psychiatric disorders.

Risperidone and quetiapine are notable in that they are the only antipsychotic medications that did not show an association with exceeding the maximum daily doses recommended by the BNF when prescribed as part of a polypharmacy approach. Furthermore, quetiapine is uniquely linked to a very low daily allowable dose when used in monotherapy. This trend can be explained by examining the prescribing practices for these medications; risperidone is prescribed at minimal effective doses 35% of the time, while quetiapine is at 48%. In contrast, olanzapine is administered within this range only 25% of the time. These findings correlate with the evidence indicating a moderate to high risk of adverse effects associated with olanzapine, while risperidone and quetiapine appear to carry lower risks. This emphasizes the importance of keeping patients on the minimum tolerable effective dose of antipsychotics, as it is essential for optimizing their treatment outcomes and minimizing the potential for adverse effects [60,61]. Continued focus on this practice will enhance patient safety and overall effectiveness in psychiatric care.

Anticholinergics are commonly prescribed for schizophrenia patients. This medication class plays a role in increasing the complexity of the prescriptions and increasing the severity of side effects, such as deteriorating patients’ cognitive functions [62,63]. Anticholinergics in our study were prescribed for 19% of patients. Although this number is less than that of other studies, which was around 30% [64,65], this number still reflects an inappropriate pattern of using anticholinergics. Recent guidelines limit the use of anticholinergics to short-term use in specific situations, such as acute dystonia and pseudo-Parkinson [11,66]. A nationwide Danish long-term study reported a reduction in prescribing anticholinergics to 5.7% over seven years after improving the clinical practice attitudes [67]. Similarly, antihistamines were prescribed for one-third of the study patients, which is considered very high when compared with (4.8%) in a recent study conducted in Qatar [68]. In general, antihistamines are not recommended by any guidelines for schizophrenia patients, as there is no sufficient available evidence to confirm their efficacy and safety [69]. Moreover, long-term use of antihistamines is not recommended and has many consequences on patients, including deterioration of cognitive functions and psychomotor side effects [70].

The concomitant use of antidepressants and APs in our research was observed in 22.2% of the studied population; this is almost threefold higher than rates reported in Japan (8.2%) [71], China (8.5%) [48], and Asia (11.7%) [64]. Add-on antidepressants to AP therapy are quite common in clinical practice for treating schizophrenia despite recent guidelines discouraging their usage [1]. Nevertheless, antidepressants may increase the probable benefits in many cases [72]. Adding antidepressants to schizophrenia patients was linked to a lower likelihood of hospitalization for psychosis and a lower mortality rate among schizophrenia patients, according to a recent real-world, long-term cohort study conducted in Finland [73].

The percentage of prescriptions containing both mood stabilizers and APs in our study was (17.7%), which is close to the rate in other studies performed in Japan (21.9%) [71], China (35.5%) [48], and Asia (13.7%) [64]. Although the wide use of mood stabilizers has been reported in different studies as an augmentation to AP for schizophrenia patients, the currently available evidence on the benefit of this practice is not convincing [74]. Based on recent systematic reviews, the results regarding this practice were mixed. Even though the risk of psychosis and schizophrenia relapse were reported to decrease, the poorer outcome and the risk of mortality rate elevation could not be eliminated [1,75,76].

Regarding the safety of using FGAP, our results are in line with previous studies that confirmed the higher severity of side effects of FGAP when compared with SGAP [77,78]. In the current study, 27.8% of patients were prescribed FGAP, which was consistent with rates reported recently in Saudi Arabia; however, prescription rates were higher when compared with those reported in other studies from Ireland (4.6%) [31], Korea (7%) [79], and Turkey (17%) [80]. The closest rate was from another study in Saudi Arabia (23.3%) [30]. This pattern is contradictory to the recent recommendations from many guidelines, which prefer using SGAP over FGAP because of the lower efficacy rate and higher severity of side effects of the latter, especially the psychomotor side effects.

Our analysis indicates that the combination of four frequently prescribed antipsychotics—olanzapine, aripiprazole, paliperidone, and haloperidol—is associated with exceeding the maximum daily allowable dose. This finding is consistent with earlier research that specifically identified olanzapine and haloperidol as contributors to surpassing recommended dosages [30]. Importantly, our study also highlights the potential for increased adverse effects associated with these medications, pointing to the necessity of careful monitoring and evaluation when treating patients with schizophrenia. This awareness can contribute to safer prescribing practices and enhance patient outcomes by ensuring effective and safe medication dosing.

One key result in our study was the high percentage (28.8%) of patients who exceeded the allowable total daily dose based on BNFmax when compared with other studies in Nigeria (8%) [81], Saudi Arabia (7%) [30], and the UK (10.1%) [82]. In addition, our data analysis showed that prescribing AP exceeding 100% of the BNFmax was significantly associated with increased severity of side effects. This association was confirmed by other studies [12,83].

This research detects many inappropriate indicators in the prescription pattern that interfere with patient safety measures. Polypharmacy with schizophrenia patients continues to be a major challenge. Many patients, especially resistant schizophrenia patients, might need more than one antipsychotic to reach the full control level. However, when add-on antipsychotic therapy is needed, we suggest limiting it to additives that have been evidentially proven to be both effective and tolerable. For example, some studies suggest adding aripiprazole for a partially responsive patient receiving a full dose of clozapine. In addition, based on our results, risperidone seems to show fewer side effects when used as an add-on. However, more extensive studies are needed to confirm this strategy.

However, many combinations should not be used, such as prescribing two FGAPs together or combining two APs having the exact mechanism of action, as this will increase the side effect severity without any therapeutic add-on efficacy for the patient. An example of this is prescribing risperidone and paliperidone together [84]. We found this practice in our analysis of nine prescriptions. We also recommend making a patient side effect assessment before considering augmentation with a second AP. If the patient has experienced a specific adverse reaction to the first prescribed antipsychotic, such as weight gain or sleep disorder, adding another drug that does not increase this side effect should be considered.

We found that 4% of the patients received a combination of a mood stabilizer and an antidepressant in addition to their antipsychotic therapy. These two groups of drugs have opposite mechanisms of action, while clinically and practically, there is no evidence suggesting this practice or mentioning a possible benefit from it [1].

In addition to controlling schizophrenia symptoms, appropriate management [85] is essential for improving social functionality and overall quality of life [86]. When adding another antipsychotic, the doses of both new and old drugs should be recalculated to avoid exceeding the maximum daily recommended dose. Prescribing more than 100% BNFmax will not offer the patient any additional therapeutic benefits [87]. A promising strategy to ensure that the patient will be maintained at lower dosages than oral AP is the well-established practice of prescribing long-acting injectable antipsychotics, which also plays a crucial role in enhancing patient compliance and reducing the risk of long-term readmission [88,89]. As previously mentioned, evidence on the benefits of antipsychotic polypharmacy for schizophrenia patients is limited, so if it should be used—in resistant cases—it must be limited to only two antipsychotic combinations, not more. Another suggestion is avoiding prescription complexity; some medications that are commonly used for schizophrenia patients without clear evidence should be withdrawn. This will improve patient safety and adherence to the treatment care plan. Regular side effect assessment should be implemented for all schizophrenia patients, especially if they are prescribed more than one medication.

The findings of this study may be subject to certain limitations. This study is based only on patients’ prescriptions provided to the hospital’s pharmacy. This may exclude schizophrenic patients who do not require regular follow-ups, resulting in an underrepresentation of the total number of schizophrenic patients recorded in the hospital’s files. In addition, it may add selection bias, thus limiting the findings’ generalizability. The size of this study’s sample may be viewed as limited, affecting the statistical power and capacity to identify unusual or less prevalent side effects. Adverse effect data are gathered through interviews with patients utilizing the GASS questionnaire. However, depending only on self-reporting increases the risk of recollection bias or false reporting of adverse occurrences. Patients may underreport or forget specific side effects, resulting in incomplete and inaccurate data. This study utilized a cross-sectional design, which may limit the establishment of causality since these data were collected at a single time point, making it difficult to determine the direction of the association. Based on available data, some patient confounders were detected, while others were absent, which may result in misleading connections. Additionally, reverse causation adds complexity to the interpretations. Other factors that could influence adverse effects, such as comorbidities, concomitant drugs, or lifestyle factors, have not been included in this study. Carrying out longitudinal studies would provide a deeper insight into prescription patterns.

## 5. Conclusions

Our study outlined many inappropriate practices in the management of schizophrenia patients. Antipsychotic polypharmacy is still commonly seen in practice despite a lack of evidence to support this practice. Also, exceeding the allowable AP daily dose is another challenge, increasing the risk of side effects without any additional effectiveness. By following evidence-based guidelines and revising prescription appropriateness with periodic assessment of the patient’s side effects should be implemented as a practice to maximize patient safety while maintaining the efficacy of the selected treatment regimen.

## Figures and Tables

**Table 1:** Patients’ baseline characteristics (*n* = 198).

Characteristic	Mean (SD)	N (%)
Age mean (SD)	40.1 (12.7)	
Sex (Male)		116 (58.6)
History of present illness		
Less than 10 years		97 (48.9)
More than 10 years		101 (51)
Comorbid diseases		
Diabetes		27 (13.6)
Hypertension		31 (15.7)
Other diseases		7 (3.5)
Glasgow Antipsychotic Side-Effect Scale (GASS) mean (SD)	16.9 (9.3)	
GASS interpretation		
Absent/mild side effects		131 (66.2)
Moderate/high side effects		67 (33.8)
BNFmax doses, mean (SD)	0.75 (0.49)	
BNFmax categories		
≤0.5		85 (42.9)
0.51–0.99		57 (28.8)
≥1		56 (28.3)
Polypharmacy		103 (52)
No of prescriptions with FGAP		55 (27.8)
No of prescriptions with SGAP		181 (91.4)
No of prescribed AP per prescription (only the antipsychotics)		
One AP		100 (50.51)
Two AP		79 (39.9)
Three AP		16 (8.1)
Four AP		3 (1.5)
Number of all psychotropic medications per prescription (antipsychotics, mood stabilizers or antidepressants)		
One medication		56 (28.3)
Two medications		93 (47.0)
Three medications or more		49 (24.7)

(SD) Standard Deviation, (GASS) Glasgow Antipsychotic Side-effect Scale, (BNFmax) British National Formulary Maximum Daily Dose, (FGAP) First-generation antipsychotics, (SGAP) Second-generation antipsychotics, (APs) Antipsychotics.

**Table 2:** Antipsychotics prescribing pattern, describing the events where each drug was prescribed as monotherapy or in combination with other antipsychotics. All percentages are out of the total number of patients included (*n* = 198).

	Prescribed as Monotherapy (No. of Prescriptions)	%	Prescribed in Combination with Other AP (One or More)	%	Total	%
Risperidone	31	15.7	41	20.7	72	36.36
Olanzapine	27	13.6	43	21.7	70	35.35
Haloperidol	12	6.1	28	14.1	40	20.20
Aripiprazole	13	6.6	23	11.6	36	18.18
Quetiapine	5	2.5	28	14.1	33	16.67
Paliperidone	7	3.5	25	12.6	32	16.16
Chlorpromazine	3	1.5	13	6.65	16	8.08
Clozapine	1	0.5	7	33.5	8	4.04
Amisulpride	-	-	7	3.5	7	3.5
Sulpiride	-	-	2	1	2	1
Flupentixol			1	0.5	1	0.5
Trifluoperazine	1	0.5	-	-	1	0.5

**Table 3:** Glasgow Antipsychotic Side-effect Scale details (the side effects arranged from the most common in descending order).

Adverse Effect	Frequency of Adverse Effect (Total *n* = 198)	%
Sedation	117	59.1
Polyuria/polydipsia	113	57
Asthenia	97	49
Tremor	97	49
Increased weight	96	48.5
Akathisia	89	44.9
Hypokinesia	84	42.4
Dystonia	74	37.4
Reduced salivation	72	36.3
Nausea/vomiting	66	33.3
Orthostatic hypotension	64	32.3
Palpitations	62	31.3
Hyperkinesia	57	28.7
Increased salivation	53	26.7
Accommodation	49	24.7
Nocturnal enuresis	40	20.2
Urinary dysfunction	34	17.2
Menorrhagia	28	14.1
Gynecomastia	20	10.1
Erectile dysfunction	15	7.5
Sexual dysfunctions	13	6.5
Galactorrhea	8	4

**Table 4:** Binary logistic regression for factors associated with having moderate/high GASS score.

GASS	*p*-Value	Odds Ratio	95% CI	
			Lower	Upper
Age	0.827	1.00	0.98	1.03
Sex	0.179	1.54	0.82	2.87
Polypharmacy	<0.001	3.21	1.64	6.29
Total number of medications per prescription	0.004	1.67	1.18	2.36
FGAP	0.03	2.79	1.11	7.01
SGAP	0.051	2.20	1.00	4.84
Presence of inappropriate frequency medication	0.718	0.88	0.44	1.76
AP dose titration	0.26	1.51	0.74	3.08

(GASS) Glasgow Antipsychotic Side-effect Scale, (FGAP) First-generation antipsychotics, (SGAP) Second-generation antipsychotics, (APs) Antipsychotics.

**Table 5:** Binary logistic regression for factors associated with having a BNFmax score of 100% or more.

	*p*-Value	Odds Ratio	95% CI	
			Lower	Upper
Age	0.155	1.0	1.0	1.0
Sex	0.741	1.1	0.6	2.3
Polypharmacy	<0.001	10.5	4.8	23.2
FGAP	<0.001	5.5	2.4	12.2
SGAP	<0.001	7.4	3.9	14.0
Presence of inappropriate frequency medication	0.478	0.8	0.4	1.6
AP dose titration	0.001	3.7	1.6	8.2

(BNFmax) British National Formulary Maximum Daily Dose, (FGAP) First-generation antipsychotics, (SGAP) Second-generation antipsychotics, (APs) Antipsychotics.

**Table 6:** Association of having a BNFmax score of 100% or more or moderate/high GASS score with different AP agents if prescribed as monotherapy or in combination.

		GASS				BNF			
		*p*-Value		95% CI		*p*-Value		95% CI	
				Lower	Upper			Lower	Upper
Olanzapine	Monotherapy	0.493	1.73	0.36	8.35	0.958	0.96	0.20	4.62
	Combination	0.009	3.46	1.36	8.79	0.011	4.13	1.39	12.34
Risperidone	Monotherapy	0.85	1.17	0.24	5.79	0.998	0.00	0.00	0.00
	Combination	0.054	2.51	0.99	6.40	0.104	0.45	0.17	1.18
Paliperidone	Monotherapy	0.993	1.01	0.09	12.02	0.645	0.56	0.05	6.65
	Combination	0.157	2.23	0.74	6.76	<0.001	15.39	3.58	66.13
Haloperidol	Monotherapy	0.451	2.02	0.32	12.63	0.999	0.00	0.00	0.00
	Combination	0.032	3.22	1.11	9.32	0.032	3.69	1.12	12.18
Aripiprazole	Monotherapy	0.923	1.10	0.15	7.98	0.219	2.88	0.53	15.51
	Combination	0.611	1.35	0.43	4.26	0.005	7.48	1.83	30.55
Quetiapine	Monotherapy	0.192	3.35	0.54	20.68	0.999	0.00	0.00	0.00
	Combination	0.111	1.94	0.86	4.37	0.775	0.85	0.27	2.63

(GASS) Glasgow Antipsychotic Side-effect Scale, (BNFmax) British National Formulary Maximum Daily Dose, (APs) Antipsychotics.

## Data Availability

Data presented in this study are available on request from the corresponding author because of ethical and privacy concerns for the patients.

## Abbreviations

The following abbreviations are used in this manuscript:

BNFmax	British National Formulary Maximum Daily Dose
GASS	Glasgow Antipsychotic Side-effect Scale
FGAP	first-generation antipsychotics
SGAP	second-generation antipsychotics
AP	antipsychotics
